# Case report: A novel R246L mutation in the LMX1B homeodomain causes isolated nephropathy in a large Chinese family

**DOI:** 10.1097/MD.0000000000037442

**Published:** 2024-03-08

**Authors:** Xian Li, Jiaojiao Fan, Rong Fu, Ming Peng, Jujie He, Qiufang Chen, Shujing Wang, Chong Chen

**Affiliations:** a Xinxiang Medical Univeisity, Xinxiang, China; b Puyang Oilfield General Hospital, Puyang, China; c The First Affiliated Hospital of Zhengzhou University, Zhengzhou, China; d Beijing Chigene Translational Medicine Research Center Co., Ltd, Beijing, China.

**Keywords:** hereditary nephropathy, homeodomain, LMX1B gene, nail patella syndrome, nail-patella-like renal disease

## Abstract

**Background:**

Genetic factors contribute to chronic kidney disease (CKD) and end-stage renal disease (ESRD). Advances in genetic testing have enabled the identification of hereditary kidney diseases, including those caused by LMX1B mutations. LMX1B mutations can lead to nail-patella syndrome (NPS) or nail-patella-like renal disease (NPLRD) with only renal manifestations.

**Case presentation:**

The proband was a 13-year-old female who was diagnosed with nephrotic syndrome at the age of 6. Then she began intermittent hormone and drug therapy. When she was 13 years old, she was admitted to our hospital due to sudden chest tightness, which progressed to end-stage kidney disease (ESRD), requiring kidney replacement therapy. Whole-Exome Sequencing (WES) results suggest the presence of LMX1B gene mutation, c.737G > T, p.Arg246Leu. Tracing her family history, we found that her father, grandmother, uncle and 2 cousins all had hematuria, or proteinuria. In addition to the grandmother, a total of 9 members of the family performed WES. The members with kidney involved all carry the mutated gene. Healthy members did not have the mutated gene. It is characterized by co-segregation of genotype and phenotype. We followed the family for 9 year, the father developed ESRD at the age of 50 and started hemodialysis treatment. The rest patients had normal renal function. No extra-renal manifestations associated with NPS were found in any member of the family.

**Conclusions:**

This study has successfully identified missense mutation, c.737G > T (p.Arg246Leu) in the homeodomain, which appears to be responsible for isolated nephropathy in the studied family. The arginine to leucine change at codon 246 likely disrupts the DNA-binding homeodomain of LMX1B. Previous research has documented 2 types of mutations at codon R246, namely R246Q and R246P, which are known to cause NPLRD. The newly discovered mutation, R246L, is likely to be another novel mutation associated with NPLRD, thus expanding the range of mutations at the crucial renal-critical codon 246 that contribute to the development of NPLRD. Furthermore, our findings suggest that any missense mutation occurring at the 246th amino acid position within the homeodomain of the LMX1B gene has the potential to lead to NPLRD.

## 1. Introduction

Nail patella syndrome (NPS), also known as hereditary nail bone dysplasia, is a rare autosomal dominant disorder, with an incidence rate of one in 50,000. The typical manifestations of patients are dysplastic nails, absent or hypoplastic patellae, elbow dysplasia, and iliac horns. About 40% of patients may have renal impairment, 10% to 12% of patients have glaucoma, and another 4% to 7% have ocular hypertension.^[1]^ The severity of renal impairment is the main factor affecting the prognosis and quality of life of patients.^[2]^

NPS arises due to mutations in the LIM homeobox transcription factor 1 beta (LMX1B) gene, which encodes a transcription factor belonging to the protein homeodomain family. LMX1B plays a crucial role in embryonic development and is expressed in various tissues. Its functions include facilitating dorsal-ventral patterning, promoting differentiation in the anterior eye region, contributing to the development of specific neuronal populations in the central nervous system, and ensuring the differentiation and proper functioning of podocytes.^[1,3]^

However, it should be noted that a subset of patients do not exhibit any abnormalities in their nail, bone, and eye, but rather solely present with isolated nephropathy, which is referred to as nail-patella-like renal disease (NPLRD). Currently, the existing literature has documented 5 distinct forms of LMX1B gene mutation that are responsible for causing NPLRD, all of which are situated within the homeodomain. Here we describe a Chinese family with autosomal dominant nephropathy found to have a novel homeodomain mutation providing further insight into LMX1B-associated kidney disease.

## 2. Ethics approval

The study was approved by the Ethics Committee of Puyang Oilfield General Hospital affiliated to Xinxiang Medical College.

The authors confirm that “written informed consent” was obtained from the patient’s legal guardian. Informed consent was obtained from the patient for publication of this case report details.

## 3. Case report

### 3.1. Clinical evaluation

Medical records were reviewed to collect clinical data including age of onset, presence of hematuria or proteinuria, kidney biopsy findings, progression to end-stage renal disease (ESRD), and extra-renal manifestations. Physical examinations evaluated for features of NPS such as nail, skeletal, eye abnormalities.

### 3.2. Kidney biopsies

Percutaneous ultrasound-guided kidney biopsies were performed on 3 affected members. Samples were evaluated by light microscopy using hemotoxylin-eosin, periodic acid-Schiff, Masson’s trichrome and silver methenamine staining. Immunofluorescence microscopy examined no immunoglobulin and complement deposition.

### 3.3. Whole-Exome Sequencing

After obtaining informed consent, peripheral blood samples were collected from 8 family members, including 5 affected individuals and 3 unaffected relatives. Whole-Exome Sequencing (WES) was performed gene company.

### 3.4. The proband

**Figure 1.**
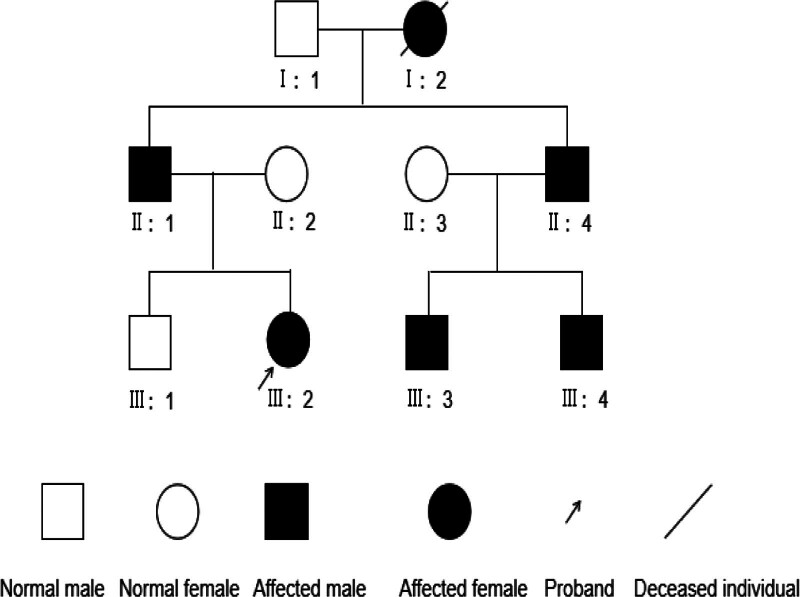
Pedigree of the Chinese family showing autosomal dominant inheritance of kidney disease.

As shown in Figure 1, the proband (III: 2) is a 13-year-old female who was diagnosed with nephrotic syndrome at the age of 6 due to edema. After taking oral prednisone and traditional Chinese medicine consecutively for about 14 months, the presence of proteinuria persisted. Subsequently, she stopped medication treatment. In August 2011 (13 years old), she was admitted to our hospital for sudden chest tightness and edema. Laboratory investigations revealed the following: urinalysis showed degree of proteinuria was 1+ to 2+; red blood cell count 5 to 8/HP; glomerular filtration rate, 7.59 mL/(min.1.73 m^2^). Blood biochemical examinations showed creatinine was 632.7 µmol/L (59–104 µmol/L); carbamide, 50.13 mmol/L (2.9–8.2 mmol/L); carbon dioxide-combining power, 14.2 mmol/L (23–30 mmol/L); blood potassium, 5.5 mmol/L (3.3–5.3 mmol/L); blood calcium, 1.43 mmol/L (2.2–2.75 mmol/L); albumin, 31.5 g/L (40–50 g/L); B-type natriuretic peptide, 5000 ng/L (<100 ng/L); blood routine showed white blood cell count was 10.8 × 10^9^ (3.5–9.5 × 10^9^), red blood cell count, 2.08 × 10^12^ (4.5–5.5 × 10^12^); hemoglobin, 31.5 g/L (131–172 g/L); Antinuclear antibody showed negative.

She was diagnosed with chronic kidney disease stage 5, renal anemia, metabolic acidosis, heart failure, hyperkalemia, hypocalcemia, and received hemodialysis at our hospital. Her kidney biopsy contained twelve glomeruli, which all showed ischemic sclerosis, diffuse atrophy of renal tubules with protein tubules visible inside. Renal interstitial fibrotic lesions were with diffuse infiltration of lymphocytes and monocytes. The glomerular arteriole walls were thickened, the lumen was narrow, and the intima was hyaline degeneration. Immunohistochemistry showed negative staining for anti-human IgG, IgA, IgM, C3, C1q on the glomerular basement membrane (GBM). Considering that the proband was young and seriously kidney disease, the pathogenic factors might be related to gene mutation, whole blood samples were collected for WES analysis after obtaining the consent of her guardian. However, the condition of the proband progressed rapidly and WES analysis was not able to perform in time.

### 3.5. Family members of the proband

#### 3.5.1. Clinical history of kidney disease.

At the same time, the family history of kidney disease was traced. It turned out that her father (II:1) had a history of proteinuria, grandmother (I:2) had a history of hypertension and an abnormal urinalysis. Routine assessment was performed on the family members. The results showed that her father, grandmother, uncle and his 2 sons all had proteinuria with/without hematuria. Her mother, brother, aunt, and grandfather of the proband all had normal urinalysis. A kidney biopsy of her father was performed and revealed focal segmental glomerular sclerosis (non specific) with ischemic renal impairment. He was treated with glucocorticoids and angiotensin converting enzyme inhibitor, and the degree of proteinuria did not decrease. A kidney biopsy of grandmother demonstrated mild glomerulopathy with ischemic renal impairment, with normal renal function and no treatment was taken.

### 3.6. Disease progression

After 9 years of follow-up, her father (50 years old) progressed to ESRD and received the initiation of hemodialysis. Her uncle (40 years old) has reached the nephrotic-range proteinuria, and was treated with oral angiotensin converting enzyme inhibitor, urinalysises of his 2 sons (10 and 12 years old respectively) both still showed microalbuminuria, and were not treated. All 3 had normal renal function.

### 3.7. Genetic analyses

**Figure 2.**
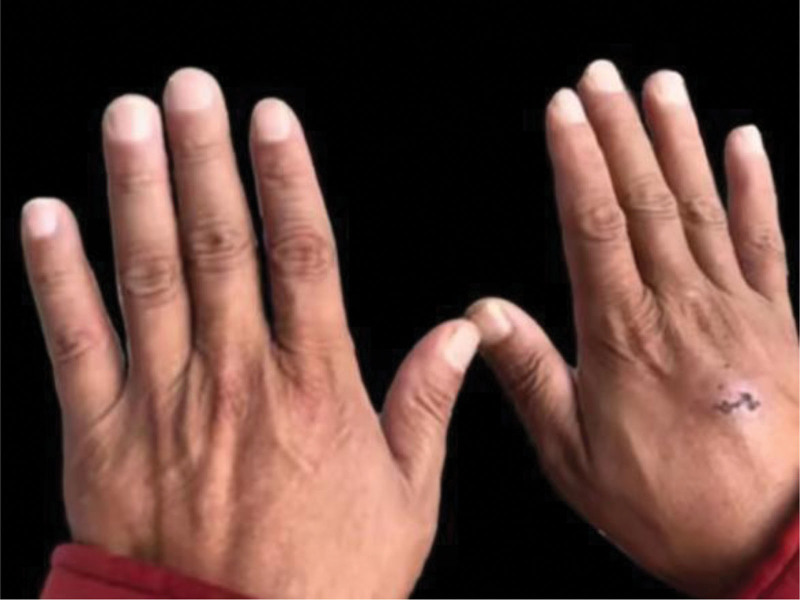
Photograph of the hands of the father (II:1), the nails of both hands are as shown in the picture, without any abnormal manifestations.

With the informed consent of the family members, a total of 9 people except the grandmother performed WES analysis. The whole blood specimen of the proband was well preserved. After the death of the proband, her father was the first to perform WES analysis in 2011. The result showed that her father had a mutation in the LMX1B gene, with a mutation form of c.737G > T, p.R246L. Upon reviewing the relevant literature, it was found that kidney disease caused by LMX1B gene mutation exists only in NPS with extrarenal manifestations. However, after detailed physical examination of members of this family, no abnormal manifestations suggestive of NPS were found, and no isolated kidney disease due to LMX1B gene were reported. The hands of the father of the proband are shown in Figure 2.

**Figure 3.**
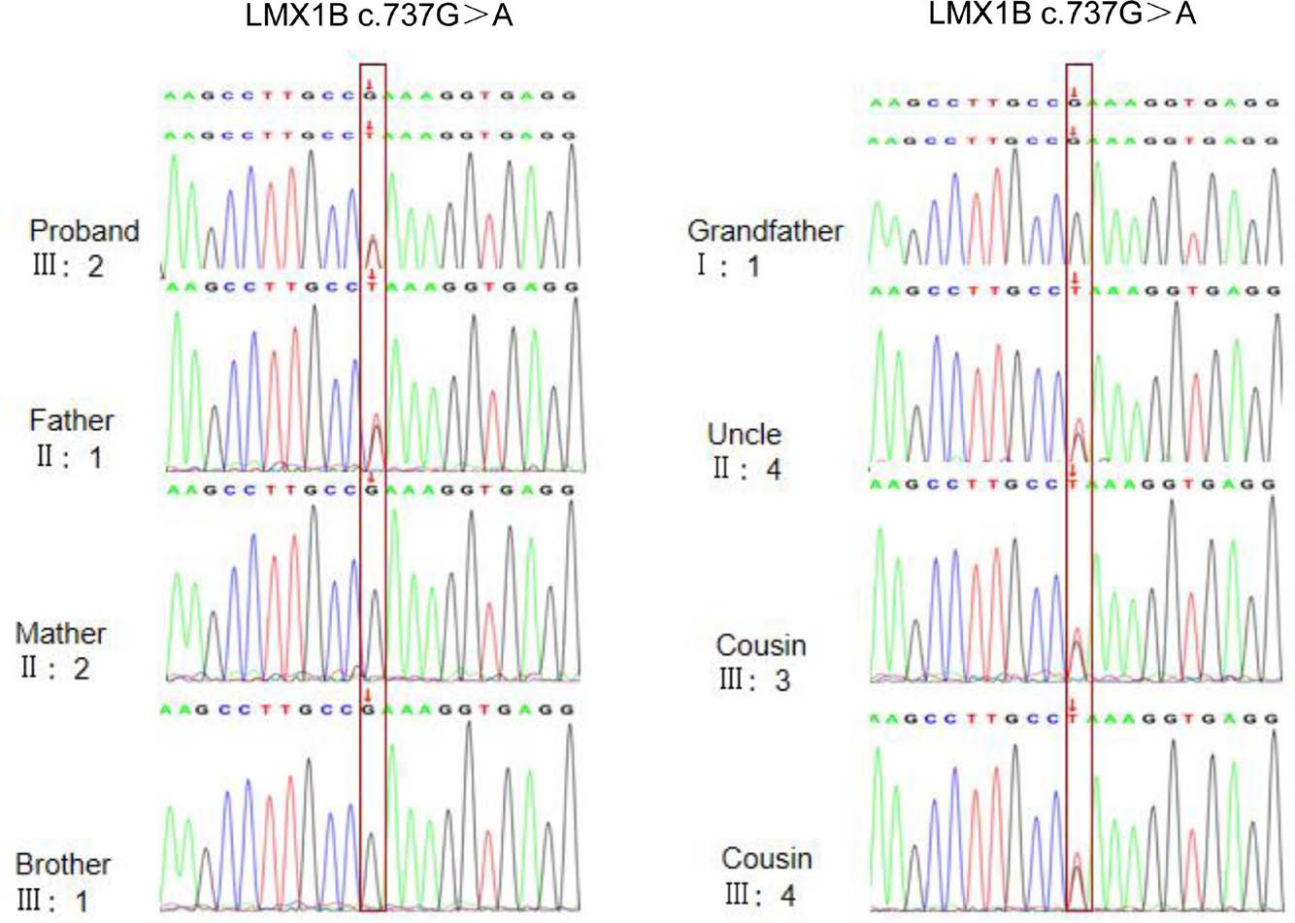
Sequence analysis of LMX1B gene in 8 members of this family. The up row is the DNA sequence of wild type, the next row of mutations. The nucleotide exchange is marked by an arrow. LMX1B = LIM homeobox transcription factor 1 beta.

**Figure 4.**
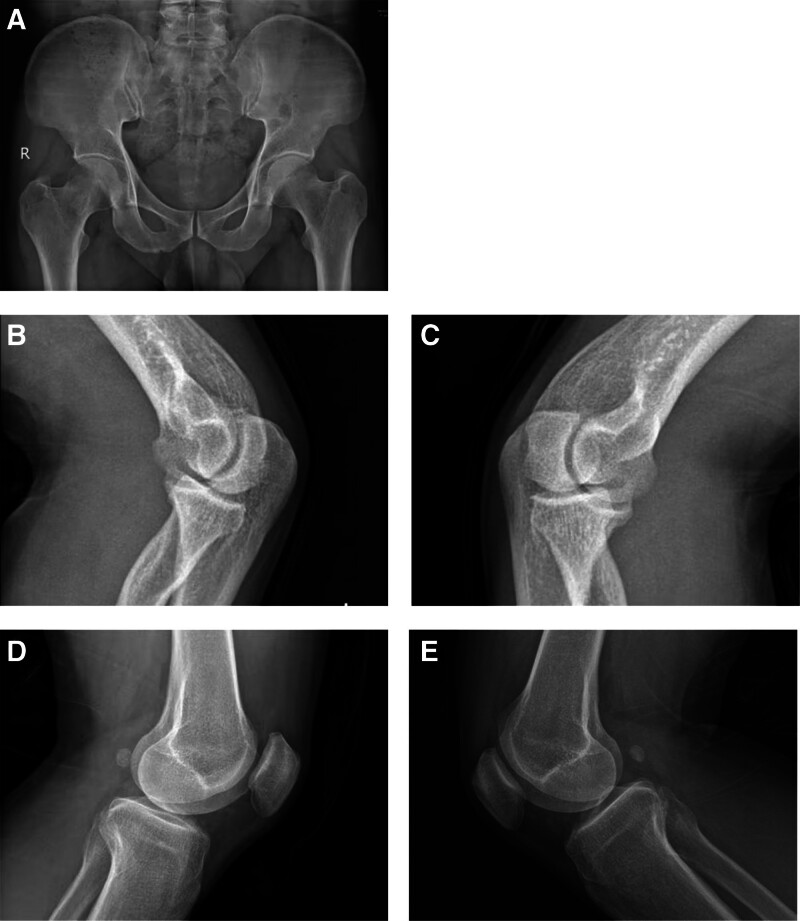
Radiographs of the pelis, elbows, and knees of the uncle (II:4), A is the pelvis of patient II: 4, B and C are the left and right elbow joints of patient II: 4, and D and E are the left and right knee joints of patient II: 4, respectively.

Combined with clinical data, we found that the kidney disease in this family was passed down from generation to generation, and there is no difference in the incidence and severity of the disease between men and women, as indicated kidney disease in this family is inherited in an autosomal-dominant fashion. As the whole blood sample of the proband was well preserved, the WES analysis was performed on her and other family members in 2020. The results of this test indicated that the proband’s uncle and 2 cousins had the same mutation as her father, namely LMX1B (c.737G > T, p.R246L). The mutant gene was absent from unaffected family members (including the 88-year-old grandfather). The gene sequences of the 8 members of the family are shown in the Figure 3. The statistical chart of population distribution of gene mutation results is shown in Table 1. The forward primer and reverse primer of cDNA of LMX1B gene were GGAGAATGCACATGCTTCCG and GGGTATGATGGGATGTGCGG, respectively. The grandmother died of lung cancer at the age of 76, and genetic testing could not be performed, but her renal function remained normal. In 2021, relevant eye examination was conducted on the father and cousins of the proband, and no abnormalities were found, excluding glaucoma, cataracts, and corneal abnormalities. Her uncle underwent bilateral knee and elbow joint X-ray and pelvic X-ray examinations (Fig. 4), and no abnormalities were found. Developmental abnormalities in nail, elbows, patella, and iliac angle were excluded.

**Table 1:** Population distribution statistics table.

Database	dbSNP	Thousand Genomes	Genome AD	Genome AD East Asia
MAF	Not included	Not included	Not included	Not included

## 4. Results

This study presents a comprehensive analysis of a large three-generation Chinese family, wherein a clear autosomal dominant pattern of kidney disease was observed. The hypothesis put forth suggests that the nephropathy observed in this family is likely attributed to a mutation in the LMX1B gene. Notably, kidney involvement was observed in 2 female and 4 male members across 3 consecutive generations. We followed the family for 9 years and did not find any extrarenal manifestation suggestive of NPS. So far, 1 female and 1 male have progressed to ESRD at the ages of 13 and 50, respectively. The rest, showed only varying degrees of proteinuria or hematuria, and their renal function remained normal. There was no significant difference in the severity of kidney involvement between male and female, consistent with the autosome-dominated genetic pattern. Table 2 presents the status of 6 patients exhibiting renal involvement. Among the total of 6 individuals, 5 have available DNA samples and display kidney involvement. WES analysis revealed that all 5 individuals possess a missense mutation in the LMX1B gene, specifically the c.737G > T, p.R246L mutation variant. Conversely, the unaffected individuals do not carry this mutant gene. Furthermore, the clinical examination did not identify any extrarenal manifestations of NPS. It is noteworthy that the mutation variant observed in this gene has not been documented in current literature and represents a novel mutation site, absent from databases such as 1000 Genomes (http://www.1000genomes.org/), Exome Variant Server (http://evs.gs.washington.edu/EVS/) or the Exome Aggregation Consortium” (ExAC) data set with exome sequence data obtained from 63,352 individuals (http://exac.broadinstitute.org/).

**Table 2:** The clinical characteristics of the affected members of this kidney disease family.

Patient	Gender	Neclcotide Alteration	Predicted Effect on Protein	Proteinuria	Hematuria	Renal pathological Diagnosis	ESRD
I:2	Female	Not detected	Not detected	2–3+		Ischemic Kidney Injury	
II:1	Male	c.737G > T	R246L	3+	2–17/HP	Ischemic Kidney Injury	50 years old
II:4	Male	c.737G > T	R246L	3+	2–4/HP		
III:2	Female	c.737G > T	R246L	3+	5–20/HP	Ischemic Kidney Injury	13 years old
III:3	Male	c.737G > T	R246L	1+–2+	2–12/HP		
III:4	Male	c.737G > T	R246L	1+	0–1/HP		

## 5. Discussion

Since the initial report by Dreyer et al that LMX1B mutations can cause NPS, research on this gene has expanded.^[2,4–6]^ NPS involves nail and skeleton defects, with about 20% of patients developing nephrotic syndrome and 10% progressing to ESRD,^[7]^ posing a major threat to quality of life.^[8,9]^ In recent years, NPLRD without extrarenal manifestations caused by LMX1B gene mutation has aroused the interest of workers.^[10–20]^ NPLRD caused by LMX1B mutations lacks extra-renal manifestations, it is often misdiagnosed due to nonspecific symptoms and renal pathology. This study presents a comprehensive analysis of a large three-generation Chinese family exhibiting an autosomal dominant pattern of chronic kidney disease. WES revealed a novel missense mutation in LMX1B (c.737G > T, p.Arg246Leu) that co-segregated with disease status, suggesting it is the underlying pathogenic variant.

In 2013, Olivia Boyer et al^[10]^ reported for the first time that 2 new mutations of the LMX1B gene, namely R246Q and R246P, could cause isolated nephropathy in 3 unrelated families in Europe, which provided us with a deeper understanding of the LMX1B gene. The clinical presentations of LMX1B gene mutation involving the kidney are complex and diverse. Patients seek medical advice for a variety of reasons, and the degree of renal involvement varies. Affected patients will present proteinuria at all ages, with or without hematuria, and can rapidly progress to ESRD in a short time. It has also been reported that some patients may present long-term proteinuria but the renal function remains normal. There is a significant difference in the severity of renal involvement in both classic NPS and NPLRD patients, and this characteristic has been described in relevant literature.^[2,10,21]^

The specific factors that influence the progression of renal function are still unknown and the clinical course of the nephropathy cannot be predicted. For example, Olivia Boyer et al reported in 2013 on a kidney disease family involving 10 members. Among them, 5 members were performed next-generation sequencing. The results showed the presence of LMX1B gene mutation, with the mutation form being c.737G > A, p.R246Q. The earliest and latest age at which family members involved in kidney disease developed ESRD were 36 and 70 years old, respectively. The remaining members also gradually developed ESRD around age 50. Noel Edwards et al^[12]^ reported in 2015 that another new form of mutation in the LMXB gene, R249Q, can cause NPLRD. There were 7 patients with kidney disease in this family for 4 successive generations, and no extrarenal manifestations suggestive of NPS were eventually diagnosed as NPLRD by Sanger sequencing, and the mutation was not present in the unaffected family members. None of the unaffected family members carried the mutation. The proband presented proteinuria at the age of 15 years, and progressed to nephrotic-range proteinuria and ESRD at the age of 28. One of the siblings presented persistent proteinuria at the age of 16, with normal renal function. At the age of 29, she developed hypertension and nephrotic-range proteinuria, who progressed to ESRD at the age of 40. Proteinuria was identified in another sibling at the age of 53, during her workup as a potential living-related kidney donor for proband. Her renal function was normal. In a family reported by Suramath Isaranuwatchai et al^[22]^ in 2021, there were 4 patients with kidney disease in 3 generations, and the proband progressed to ESRD at age 33. His son was diagnosed with steroid-resistant primary FSGS at age 8 and peritoneal dialysis was initiated at age 15. The mother of proband died at the age of 30 from complications of kidney disease of unknown cause. His brother developed ESRD at the age of 49. WES analysis presented that LMX1B (c.737G > A, p.R246Q) mutation existed in all patients. In 2022, Chinese scholar Wang Fang et al^[23]^ reported a family of hereditary kidney disease with asymptomatic proteinuria as the prominent manifestation. The proband was 11 years old and presented with hematuria with nephrotic-range proteinuria and normal renal function. The mother of the proband had a history of chronic nephritis for 9 years. Laboratory examination at the age of 46 indicated the presence of hematuria and nephrotic-range proteinuria. Her 24-year-old sister showed only hematuria and microalbuminuria. All 3 had no abnormalities in their nails, limbs or joints. Sanger sequencing confirmed that LMX1B (c.737G > A, p.R246Q) is the pathogenic gene for their kidney disease.

Five members of the family we reported have been confirmed to had the same missense mutation, c.737G > T (p.R246L), in LMX1B. It is characterized by co-segregation of genotype and phenotype. There are significant differences in the severity of kidney disease among different members. Two of them developed ESRD at the ages of 12 and 49. The grandmother of the proband, who presented proteinuria and died of a lung tumor at age 76. Her renal function had been in the normal range. These characteristics exist in previously reported NPS and NPLRD.

The kidney biopsy manifestations of NPLRD patients under light microscopy are diverse and nonspecific, with most presenting as focal segmental glomerulosclerosis (FSGS). Immunofluorescence staining is negative for IgG, IgM, and IgA. Some patients can show characteristic manifestations revealed by electron microscopy, focal or diffuse irregular thickening of the GBM, with electron-lucent areas (“moth-eaten appearance”) and the deposits of type III collagen fibrils bundles in the basement membrane_._^[19]^ The GBM is in dynamic change, and this characteristic may appear under the electron microscope over time.^[23]^ These renal pathological manifestations are consistent with those of patients with NPS involving the kidneys.^[2]^ However, the characteristic manifestations of electron microscopy can also appear in patients without renal manifestations.^[24]^ Recent research reports have indicated that the characteristic electron microscopy findings of Fabry, specifically myelin figures and zebra bodies, can manifest in patients with NPLRD. However, no gene mutation or abnormal enzyme activity associated with Fabry has been identified. Genetic testing has revealed that all patients possess LMX1B (c.737G > A, p.R246Q) mutations, leading to their eventual diagnosis of NPLRD.^[13,14,17]^ This discovery enhances our comprehension of kidney biopsy in NPLRD. In our reported renal disease cohort, all 3 patients who underwent kidney biopsies exhibited ischemic renal impairment, and immunofluorescence shows negative antibodies in the mesangial area of kidney biopsy.

The LMX1B gene is located on the autosome at 9q34.1 and consists of 8 exon, encoding about 372 approximately amino acids. So far, it has not been found that the same mutation can cause both NPS and NPLRD. The LMX1B gene is involved in the formation of the dorsal limb patterning, and begins to be expressed in the kidney on 14.5 days of the mouse embryo. At this time, the splanchnic cells of renal capsule are gradually differentiating into podocyte, and continue to exist throughout the embryonic development and after adulthood_._^[11]^ The LMX1B gene is essential for the development of glomerulus, and it plays a regulatory role in the expression of multiple podocyte genes. These regulated genes are critical to the differentiation and function maintenance of podocyte,^[25]^ for example, they are involved in the regulation of podocyte on the expression of collagen protein in GBM.^[26]^ The normal function of podocytes and the maintenance of the normal morphology of basement membrane are the key to ensure the normal glomerular filtration barrier. The LMX1B gene is transcribed and cleaved to form a product approximately 7 kb in size.^[27]^ Its amino terminal is 2 tandem zinc binding domains rich in cysteine, known as the LIM domain, which is responsible for the interaction between proteins.^[28]^ It has not yet been shown to bind to DNA. The carboxyl end is rich in glutamine and serine, representing the transcriptional activation domain. The homeodomain near the LIM domain is the location of binding specific DNA promoter and enhancer sequences, which can specifically bind to FLAT elements,^[25,26]^ and is necessary for DNA binding and transcriptional activation. In a 2005 paper published by Bongers et al, it was shown that patients with NPS whose mutations occurred in the homeodomain had a greater incidence of kidney involvement and a higher degree of proteinuria than patients whose mutations occurred in the LIM domain.^[29]^ Olivia Boyer et al Further highlighted the correlation between the LMX1B homeodomain and renal function in the context of isolated nephropathy studies. Currently reported typical NPS caused by LMX1B gene mutation, most mutation sites are located in LIM-A and LIM-B domain, and a few are located in homeodomain,^[28]^ emphasizing the importance of the functions of these regions. It is important to note that the mutation sites responsible for the NPLRD reported so far are all located in the homeodomain. At present, 5 missense mutations in the LMX1B homeodomain that can cause isolated nephropathy have been reported in the relevant literature. They are R246Q, R246P, R249Q, A278V, and P219A, respectively.

Combining inheritance pattern, clinical features of kidney disease, and WES analysis, we speculate that a novel mutation in the LMX1B homologous domain of R246L may be the causative gene for hereditary kidney disease in this family. This indicates a high likelihood of a missense mutation at site 246 leading to NPLRD and highlights the crucial role of arginine at position 246 in maintaining kidney function stability. In terms of amino acids, arginine is a polar amino acid with charge, leucine and proline are non-polar amino acids without charge, and glutamine is a polar amino acid without charge. Most proteins contain polar amino acids on the surface and non-polar amino acids in the interior. The uncharged amino acid side chain typically consists of alkyl, alcohol, and coolamine groups, and does not participate in any ionization reactions. Thus, in terms of the nature of the amino acid, arginine mutates into leucine, proline or glutamine, affecting the structure and function of the protein and thus causing the associated clinical symptoms.

Additional research has revealed a substantial body of literature investigating site 246,^[10,11,15]^ specifically in relation to its involvement with the kidney, thus confirming its kidney-specific nature. R246, a highly conserved element across biological evolution, is an integral component of a specific sequence and has been demonstrated to play a crucial role in the functionality of the LMX1B gene, which is essential for protein binding to the gene. The positively charged guanidine group of arginine exhibits a close affinity to the negatively charged DNA backbone,^[10]^ thereby facilitating subsequent reactions. According to the aforementioned findings, it can be inferred that the R246 mutation is likely to diminish the aforementioned interaction. Additionally, the substitution of arginine with glutamine at position 249 within the homeodomain has been observed to result in isolated nephropathy. In the silicon model devised by Noel Edwards et al, it is postulated that R249 is situated in proximity to the primary DNA strand, where the negatively charged DNA phosphorylation framework binds and interacts with one another, thereby reaffirming the significant involvement of arginine.^[12]^ Tsuyoshi Isojima et al^[19]^ explained the possible causes of NPLRD caused by R246 missense mutation. The conducted experiments provided evidence that the transcriptional activity of R246Q exhibited partial impairment. While R246Q adequately facilitated ungual and patellar development, it proved insufficient in sustaining glomerular differentiation and function. The observed disparity in phenotypes between NPS and NPLRD patients could potentially be attributed to deficiencies in the transcriptional activity of R246Q. Furthermore, the identification of residual activity mutations in typical NPS patients suggests that the manifestation or absence of extrarenal symptoms may not solely rely on the transcriptional activity of the LMX1B gene.^[5]^

The LMX1B gene plays a role in the nucleus, participates in the storage, replication and transcription of genetic information in cells, and affects the growth and development of the body.^[30,31]^ In terms of kidney, it regulates the development of embryonic kidney,^[4]^ and studies have shown that LMX1B is essential for maintaining podocyte differentiation in adult kidney.^[32]^ Mutations at sites in the homeodomain may result in the loss or inactivation of the homeodomain, making it unable to recognize and bind to the target gene and thus unable to perform its transcription function. In particular, mutations at site 246 are directly related to proteinuria, further suggesting the importance of this region for stabilizing podocyte function. In the single-cell sequencing database of healthy adult kidneys, we found that LMX1B is mainly expressed in podocyte, and it is very low or even not expressed in other cells, indicating that LMX1B gene is closely related to podocyte. Claudia Rohr et al reported that LIM-homeodomain transcription factors play a key role in podiocytes. They found that the LMX1B knockout mice had generally impaired glomerular development, reduced expression of GBM collagen, in particular, severely impaired differentiation of podiocytes without the formation of foot processes and split diaphragm.[33]

**Figure 5.**
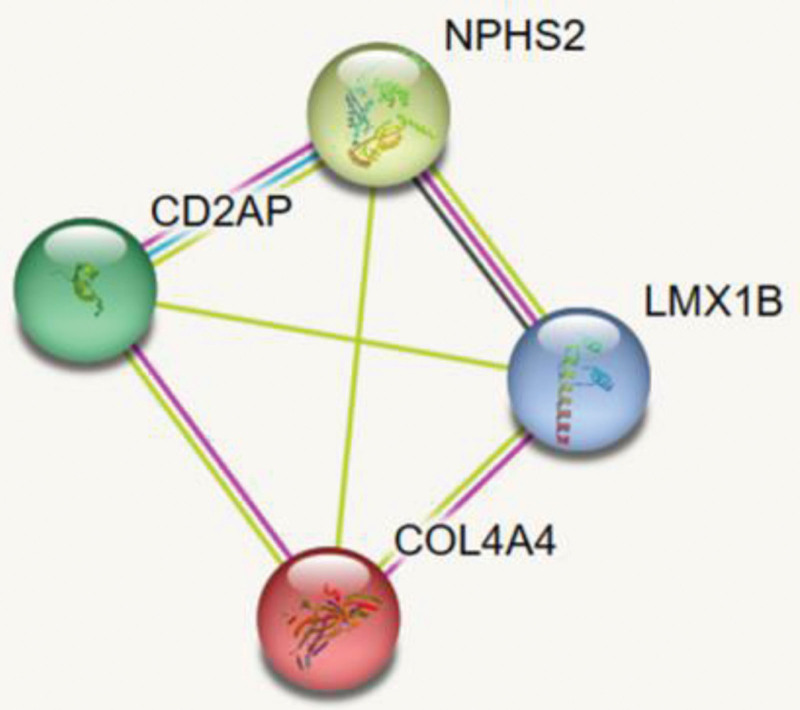
The red line represents gene fusion; The green line represents genetic affinity; The blue line represents gene co-production; The purple line represents experimental proof; The yellow line represents text mining evidence; The light blue line represents auxiliary database evidence; The black line represents gene co-expression.

Which of the split diaphragm associated molecules are regulated by the transcription factor LMX1B? We first screened through the Protein Interaction Network Database. The results showed that LMX1B and *NPHS2* have co-expression of genes, suggesting that the LMX1B might be involved in the transcription of *NPHS2* gene. It is well known that *NPHS2* gene is specifically expressed in podocin to encode podcoin. Podocin is a key protein of podocin fissure membrane protein complex and one of the important components of glomerular filtration barrier, which plays an important role in podococyte function,[34,35] while podocin dysfunction is one of the causes of proteinuria. A number of scientific research have attempted to elucidate the specific mechanism of action of LMX1B in podocytes. The NPHS2 promoter contains a cis-acting LMX1B*-FOXC* sequence that binds to both LMX1B and *FoxC2*.[36] The experiments of Claudia Rohr et al have shown that the LMX1B gene binds to two AT-rich sequences in the promoter region of the *NPHS2* gene^[33]^ for subsequent reactions. At the same time, LMX1B also maintains downstream genes encoding *CD2AP* protein.^[26]^ Both podocin and *CD2AP* proteins are essential in the selective permeability of the glomerular filtration membrane. The mRNA and protein levels of *CD2AP* and podocin in LMX1B ^*-/*-^ podocytes significantly decreased,^[25]^ indicating that these molecules play a synergistic role in the formation of foot processes and fissure membranes. The study by Jeffrey H. Miner et al found the binding site of LMX1B in the putative regulatory region of *CD2AP* and *NPHS2*, proving that LMX1B binds to these sequences in vitro and can activate transcription through them.^[25]^ Olivia Boyer et al also demonstrated that R246 in the homeodomain may be the key to podocyte specific downstream target interaction, which is important for type IV collagen α 3 α 4-chain, podocin, and *CD2AP* proteins are of great significance.^[10]^ We constructed a protein interaction network diagram (Fig. 5) for the 4 genes LMX1B, *COL4A*4, *NPHS2*, and *CD2AP*, and the results showed the direct effect of LMX1B on *NPHS2*. All of this suggests that the LMX1B gene plays an important role in the maintenance of normal kidney function.

In conclusion, our report strongly suggests that the pathogenic etiology of this hereditary kidney disease family is most likely attributed to the LMX1B (c.737G > T p.R246L) mutation. However, it is important to acknowledge that the influence of other factors on the kidney phenotype of this family cannot be definitively excluded at this time. Moreover, the renal specificity of the R246 mutation underscores the significance of the LMX1B gene, particularly its homeodomain, in the identification and transcription of specific genes. This further underscores the indispensable role of genetic testing in the accurate diagnosis of hereditary diseases.

## Acknowledgments

We sincerely thank the patients and their families who participated in this study, as well as our pediatric team.

## Author contributions

**Conceptualization:** Rong Fu.

**Data curation:** Jujie He, Qiufang Chen.

**Formal analysis:** Shujing Wang.

**Project administration:** Chong Chen.

**Writing – original draft:** Xian Li.

**Writing – review & editing:** Jiaojiao Fan, Ming Peng.
